# Advances in Cyclodextrins and Their Derivatives in Nano-Delivery Systems

**DOI:** 10.3390/pharmaceutics16081054

**Published:** 2024-08-09

**Authors:** Xin-Yu Ji, Yi-Xuan Zou, Han-Fang Lei, Yong Bi, Rui Yang, Ji-Hui Tang, Qing-Ri Jin

**Affiliations:** 1 School of Pharmacy, Hangzhou Medical College, Hangzhou 310053, China; jixinyu_0520@163.com; 2 National institute of Metrology, Beijing 100029, China; 3 College of Pharmacy, Anhui Medical University, Hefei 230032, China; leihf@shanhe01.com (H.-F.L.); by@shanhe01.com (Y.B.); 4 NMPA Key Laboratory for Quality Research and Evaluation of Pharmaceutical Excipients, National Institutes for Food and Drug Control, Beijing 100050, China; yangr@nifdc.org.cn

**Keywords:** cyclodextrin, cyclodextrin derivatives, nano-delivery systems

## Abstract

The diversity of cyclodextrins and their derivatives is increasing with continuous research. In addition to monomolecular cyclodextrins with different branched chains, cyclodextrin-based polymers have emerged. The aim of this review is to summarize these innovations, with a special focus on the study of applications of cyclodextrins and their derivatives in nano-delivery systems. The areas covered include nanospheres, nano-sponges, nanogels, cyclodextrin metal–organic frameworks, liposomes, and emulsions, providing a comprehensive and in-depth understanding of the design and development of nano-delivery systems.

## 1. Introduction

Cyclodextrin (CD) is one of the most widely used excipients produced in nature. It is a cyclic oligosaccharide formed from α-1,4-linked glucose units, where α-CD, β-CD, and γ-CD are the most common natural forms, containing 6, 7, and 8 glucose units, respectively [1]. The abundant hydroxyl groups of CD make it favorable for chemical modification [2], where these groups can be oxidized, esterified, or cross-linked with polymers to prepare CD derivatives [3]. The CD molecule also has an internal three-dimensional hydrophobic cavity, while the outside is hydrophilic [4]. The diversity of CDs and their derivatives are increasing with continuous research, from monomolecular CDs with different branched chains to CD-based polymers, such as CD-based polyrotaxanes [5], polypseudorotaxanes [6], and grafted CD polymers and cross-linked CD polymers [7]. The derivatives of CD are superior to natural CD due to their good biocompatibility, biodegradability, stimulus responsiveness, and the ability to target specific organs, making them an excellent choice as drug delivery systems [8].

CD can form inclusion complexes with hydrophobic molecules, such as paclitaxel, curcumin, catechin, quercetin, and other hydrophobic drugs [9,10,11]. This property makes it widely used in the field of nanotechnology, such as the delivery of drugs and genes [12]. With the continuous development of nanotechnology, CD and its derivatives represent an important class of nanomaterials and have shown remarkable research value in nano-delivery systems, such as micelles, nanoparticles, nanofibers, and nanorods [13].

**Figure 1:**
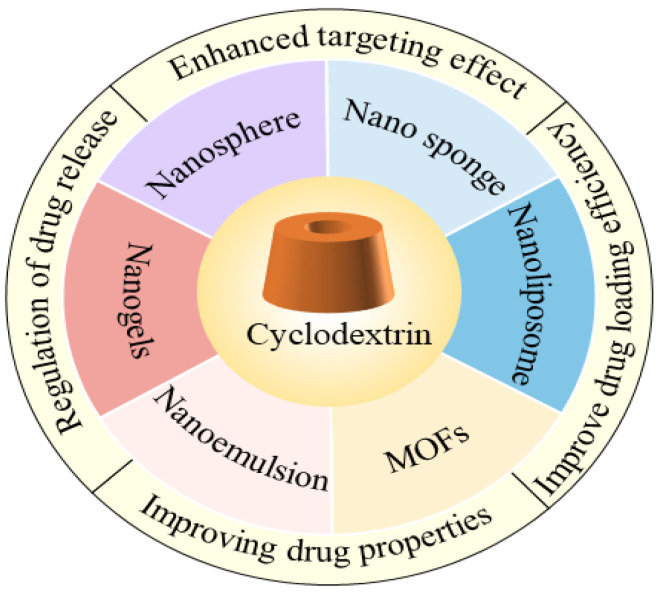
Advances in CDs and their derivatives in nano-delivery systems.

This review summarizes these innovations, with a special focus on the study of applications of CDs and their derivatives in nano-delivery systems. By covering areas such as nanospheres, nano-sponges, nanogels, CD metal–organic frameworks, liposomes, and emulsions, this review will delve into the properties of these materials as carriers and their potential applications in nano-delivery systems. This comprehensive and in-depth understanding will provide comprehensive support for the design and development of nano-delivery systems and will promote the further innovation and application of nanotechnology in biomedicine, drug delivery, and other fields. In this dynamic area of research, this review focuses on the applications of CDs and their derivatives and the prospects that they bring, providing readers with a starting point to learn more. Figure 1 summarizes the advances of CDs and their derivatives in nano-delivery systems.

## 2. Cyclodextrins (CDs)

### 2.1. The Types and Characteristics of Natural CDs

The discovery and study of CDs has a history of 130 years. In 1891, Antoine Villiers first reported on α- and β-CDs, conducting pioneering research on their composition and chemical properties [14]. CDs are a type of cyclic oligosaccharide derived from the enzymatic hydrolysis of linear starch and are composed of six or more α-1,4-linked glucose units. The typical natural CDs, namely, α-, β-, and γ-CDs, contain 6, 7, and 8 glucose units, respectively [15]. The inner diameters of the hydrophobic cavities of α-, β-, and γ-CDs are 4.7–5.3, 6.0–6.5, and 7.5–8.3 Å, respectively [16].

β-CD is more accessible, cost-effective, and has a hydrophobic cavity size suitable for encapsulating a large number of drug molecules, making it widely used in various applications [16,17]. However, the hydroxyl groups on the adjacent glucose units of β-CD have strong intermolecular interactions, meaning they do not readily interact with water molecules, leading to the relatively poor aqueous solubility of β-CD. However, α-and γ-CDs are less restricted, with weaker interactions between the hydroxyl groups of adjacent glucose molecules in the ring, which results in a higher solubility than that of β-CD [18].

The surface of CDs is rich in hydroxyl groups, but natural CDs and their complexes have limited solubility in aqueous solutions. Therefore, various derivatives have been developed, for example, by modifying and substituting methyl, ethyl, hydroxypropyl, and sulfobutyl groups, to improve the solubilization and inclusion capacity of natural CDs [16,19,20,21]. Table 1 summarizes the characteristics of three natural CDs.

### 2.2. Formation of the CD Inclusion Complex

The most significant feature of CD is the formation of inclusion complexes with various solids, liquids, and gases through host–guest interactions. Since the sizes of the host and guest molecules are compatible, the guest molecules exist within the hydrophobic cavity of the CD [17]. More specifically, CD inclusion complexes are in a dynamic equilibrium, the state of which is determined by the value of the stability constant. The higher the value of this constant, the greater the stability of the inclusion complex [25].

In an aqueous solution, due to non-covalent bonding (such as van der Waals forces and hydrogen bonds), CD forms inclusion complexes with guest molecules at a molar ratio of 1:1, but there are also types involving ratios of 1:2, 2:1, 2:2, and 3:1 [26]. The formation of inclusion complexes between drugs and CDs is dependent on a variety of factors, including the chemical structure of the drug and the selection of the type of CD, solvent, preparation method, and temperature, among others [27]. Eid et al. characterized the phase solubility of the inclusion complexes of thymoquinone (TQ) and sulfobutylether-β-CD (SBE-β-CD) at five different temperatures (293–318 K). The study found that the aqueous solubility of TQ increased linearly with the increase in the molar concentration of SBE-β-CD, indicating the formation of an inclusion complex [28].

### 2.3. Types and Characteristics of CD Polymers

CD polymers are polymers or networks containing CDs, in which CDs are the main components [7], including polyrotaxane, polypseudorotaxane, and grafted CD polymers. Polyrotaxane is a supermolecular structure obtained by the polymerization of rotaxanes through covalent bonds [16,29]. CDs play an important role in polyrotaxanes, with the most typical type being the polyrotaxane based on α-CD and polyethylene oxide [7]. For example, Yu et al. developed supramolecular nanomedicines using an amphiphilic diblock copolymer as the axle and a primary-amino-containing β-CD (β-CD-NH_2_) as the wheel, with the system being driven by the host–guest interactions between β-CD-NH_2_ and segments of poly(ε-caprolactone) [30].

Polypseudorotaxanes are very similar to polyrotaxanes, with the difference being that polypseudorotaxanes do not have end-capped macromolecules present [6]. Crivello et al. designed a poly(pseudo)rotaxane supramolecular hydrogel composed of a mixture of α-CD and two customized polyether polyurethanes (PEUs) to encapsulate lignin–cobalt nanoparticles, which was a promising system for treating chronic wounds [31]. CDs can be used to graft various linear, branched, cationic, anionic, copolymer, and cob lock polymers. Linear grafted CDs have the widest range of applications, such as chitosan [32], alginate [33], and cholesterol [34]. For example, Wang et al. used epichlorohydrin (ECH) as a cross-linker to covalently graft chitosan with β-CD (CS-g-β-CD) under mild conditions for the controlled release of the anticancer drug etoposide (VP16), which was stimuli-responsive to pH and temperature [35]. Khodayari et al. grafted hyaluronic acid/β-CD onto FeO magnetic nanoparticles for doxorubicin (DOX) delivery, and the drug release in the simulated cancer fluid (pH = 5.6) reached 92.43% after 48 h, showing a higher DOX release than at pH = 7.4 (77.05%; 48 h) [36].

## 3. CD-Based Nano-Delivery System

In recent decades, with the continuous development and progress of nanotechnology, nanocarriers have played an important role in drug delivery. They can be used as drug carriers to control drug release or play a targeted role through design or modification [37]. A variety of types of nanocarriers have been applied to drug delivery, including liposomes, nanospheres, nano-sponges, emulsion, nanogels, dendrimers, and metal–organic frameworks [38,39].

### 3.1. Nanospheres

Nanoparticles are small colloidal particles prepared from biodegradable or non-biodegradable materials, with an average diameter ranging from 10 to 1000 nm. Nanospheres are a specific type of nanoparticle that can uniformly disperse drugs within a matrix [40].

CDs have been widely used to improve the solubility of poorly soluble drugs. Natural CDs and their derivatives form inclusion complexes with these drugs, thereby increasing the drug load of the nanospheres. This allows them to carry a large amount of medication and offers advantages such as enhancing drug permeability, targeting, efficiency, and reducing side effects [41]. Miranko et al. used different CDs to alter the permeability behavior of cetirizine hydrochloride (LC) and found that CDs could significantly enhance its permeability in the nasal cavity, with β-CD notably improving permeability [42]. These researchers developed a star-shaped cationic polymer, CD-OEI, which consisted of a β-CD core and three arm-shaped ethyleneimines (OEIs) for the co-delivery of DOX and the *p53* gene. This supramolecular drug and gene co-delivery system exhibited high gene transfection efficiency and effective protein expression in vitro and reduced cell activity and enhanced antitumor effects at low DOX concentrations, showing the promise of combining drugs and genes to treat cancer [43].

Targeting drugs to specific organs can increase the accumulation of the drug, thereby improving therapeutic efficacy and reducing adverse reactions. Chen et al. designed a bio porous nanosphere loaded with sorafenib (SF) using RNA as a nano-scaffold and CD as a binder [44]. The RNA contained Epithelial-cell adhesion-molecule (EpCAM) aptamers for targeted delivery and siRNA sequences for EpCAM gene silencing, and CD could load the first-line drug sorafenib for the targeted therapy of hepatocellular carcinoma through its hydrophobic cavity. With the assistance of nucleic acid aptamers, the drug-loaded porous nano-spheres (PRS@SF) were internalized by hepatocellular carcinoma cells and degraded by the intracellular dicer enzyme to produce siRNA and release SF, which was effective for the co-treatment of hepatocellular carcinoma cells. The porous nucleic acid nanospheres, which contain nucleic acid sequences and CDs, can be used to support different types of gene drugs and small molecule drugs, respectively, providing a new pathway for the targeted delivery of appropriate therapeutic drugs to specific tumors.

### 3.2. Nano-Sponges

Nanoparticles are drug delivery systems with nanoscale dimensions, characterized by a high surface area-to-volume ratio, which exhibit enhanced permeation and retention effects at tumor sites [45]. Nano-sponges, a special type of nanoparticle delivery system, are known for their strong permeability and good biocompatibility, bioavailability, and stability. They have been widely applied in drug delivery systems and cancer therapy [46].

CD-based nano-sponges possess a sponge-like structure that can form inclusion or non-inclusion complexes with various drugs or active ingredients, serving as an effective delivery vehicle for drugs with low solubility, permeability, and bioavailability. These CD-based nano-sponges combine the highly biocompatible and biodegradable and low toxicity characteristics of CDs with the high thermal stability and insolubility of nano-sponges [47]. CD-based nano-sponges have potential applications in drug delivery systems [48].

Many drugs belong to BCS (biopharmaceutics classification system) II drugs, with low solubility and high permeability. Up to 40% of new drugs are insoluble in water, which greatly limits their clinical applications [49]. CD-based nano-sponges can be used to increase the solubility of insoluble drugs [50]. The complex of the loaded drug with CD will reduce the crystallinity of the drug and thus improve its solubility [51]. Compared with conventional CD complexes, the curcumin solubility of CD nano-sponges was higher, possibly because the capture of curcumin in the nano-sponge reduced the particle size of the drug and significantly enhanced its solubility [52]. For example, Mashaqbeh et al. investigated the complex stability and solubility of curcumin with β-CD and β-CD-based nano-sponges. Compared with the solubility of free drugs, the formation of a β-CD inclusion complex increased the solubility of curcumin by 2.34 times, while when curcumin was loaded in β-CD-based nano-sponges, the solubility increased by 2.95 times. The stability constant of the curcumin nano-sponge is 4972.90 M^−1^, which was 10 times higher than that of the β-CD complex (487.34 M^−1^) [53]. Loading active substances in nano-sponges can not only maintain the therapeutic effects but also allows for the design and development of delivery systems for different controlled drugs, which can significantly improve the compliance of patients by reducing the frequency of drug administration [54]. Dai et al. used acryloyl-6-ethylenediamine-6-deoxy-β-CD (β-CD-NH-ACy), acrylic acid (AA), and N, N-bis(acryloyl)-cystamine (BACy) as cross-linking agents, and a valley nano-sponge based on a β-CD-attached highly cross-linked polymer was developed for the controlled release of DOX, which had a high drug-loading rate (22.6%), degradability, and pH response. In vitro release studies showed that the DOX release from nano-sponges was significantly increased (~77.0%) under acidic (pH 5.0) and cytosolic-reducing (10 mM GSH) conditions [55].

### 3.3. Liposomes

Liposomes are defined as bilayer lipid vesicles with a lipid and aqueous bilayer structure, which allow them to encapsulate both hydrophilic and hydrophobic drugs while maintaining their nanometer size during storage and application [56]. Liposomes offer many advantages in drug delivery and drug targeting [57], such as biocompatibility, reduced toxicity and multidrug resistance [58], and efficient drug delivery [59]. The encapsulation of poorly soluble drugs in lipid bilayers is usually limited by the mass ratio of drug to lipid, so trapping CD inclusion complexes in liposomes can be used to overcome this shortcoming [60].

Solubility is an important factor affecting the achievement of the desired concentration of a drug in systemic circulation to produce the expected pharmacological response. Drugs with low solubility have disadvantages such as low absorption and bioavailability, frequent high-dose administration, and difficulties in development [61]. Silibinin is a natural flavonoid compound used clinically for the treatment of hepatitis. However, its lower aqueous solubility limits its bioavailability [62]. In response to this issue, researchers formed an inclusion complex of silibinin with hydroxypropyl-β-CD (HP-β-CD) and further prepared nanoliposomes. The results show that the maximum release of silibinin from nanoliposomes was 75.40% ± 0.73, while solubility was 73.95 mg/mL, and relative bioavailability was 4.52 [63]. In another study, Aloisio et al. studied the effects of β-CD, methyl-β-CD (M-β-CD), HP-β-CD, and meglumine (MEG) on the low water-soluble drugs sulfamethazine (SMR) and indomethacin (INM) and further prepared them into liposomes. The entrapment efficiency of SMR and INM for MEG and HP-β-CD liposomes were higher (5636.28 and 439.54 mmol/mol), and compared with the ligand-free preparation, the entrapment values of SMR and INM were 18 and 43 times higher, respectively [64].

When poorly soluble drugs are complexed with CD and are encapsulated in the aqueous phase of liposomes, they are provided with dual protection, thereby significantly enhancing the stability of the poorly soluble drugs. Quercetin is a dietary flavonoid with the characteristic of chemical instability. It is easily oxidized and degraded during storage, which greatly limits its application [65]. Azzi et al. encapsulated quercetin with CDs, liposomes, and CD liposomes (DCLs). This phase solubility study showed that the solubility of quercetin was enhanced after complexing with CD, and the quercetin encapsulated in DCLs was better protected from ultraviolet irradiation, and the photostability was further improved [66]. The liposome formed by the catechol–CD complex and soybean lecithin (CCPL) was stable at 4 °C for 15 days but precipitated at 37 °C, indicating that temperature is one of the key parameters affecting liposomes [67]. The stability of liposomes can be enhanced by post-processing techniques such as freeze drying, spray drying, and spray freeze drying [68].

### 3.4. Metal–Organic Frameworks

Metal–organic frameworks (MOFs) are highly ordered crystalline porous coordination polymers (PCPs), which include organic and inorganic ligands. They are formed by coordination bonds between inorganic ligands (such as metal ions or clusters) and organic ligands (such as carboxylates, phosphonates, and phenolates) to create one-dimensional/two-dimensional/three-dimensional networks of organic frameworks [69,70].

Although MOFs have the characteristics of a high specific surface area, adjustable pore structure, and good thermal stability, their inherent toxicity and moisture sensitivity limit their application [71]. Therefore, the synthesis of linkers and metal ions with good biocompatibility, such as CDs with host–guest interactions; a high encapsulation ability; and hydrophilicity, provides broader prospects for MOFs [71]. Forgan et al. have demonstrated that in the presence of alkali metal salts, γ-CD can be connected to group IA and IAA metal cations through coordination to form an MOF in a manner similar to crown ethers [72]. As a special kind of carbohydrate organic ligand, CD showed -OCCO- motifs on its primary and secondary planes, which improved the probability of forming an extended structure with metal ions. The common low-toxic or non-toxic metal ions that make up Bio-MOFs include K^+^, Ca^2+^, Zn^2+^, Fe^3+^, Zr^2+^, Cu^2+^, and so on. Potassium, as a major element in organisms, is well tolerated by the body. CD-MOF with K^+^ as the metal ion, due to its excellent drug-loading capacity and high biocompatibility, enables drug targeting and facilitates the improvement of solubility and bioavailability for insoluble drugs, thereby enhancing drug stability.

Drug treatments for acute lung injury (ALI) are unsatisfactory, because drugs cannot specifically target the lungs. Based on the typical characteristics of ALI reactive oxygen species (ROS) excess and acute inflammation. He et al. used CD-MOFs as the template and oxalyl chloride as the cross-linking agent to design a peroxyoxalate bond. The new carrier, OC-COF, was loaded with the natural drug molecule ligustrazine (LIG) to prepare a LIG@OC-COF dry powder for inhalation, which possessed a high deposition rate in the lungs [73]. The researchers used incubation methods to load paeoniflorin (PAE) particles into CD-MOF for inhaled administration to treat acute lung injury. Then, A549 and Caco-2 cell lines were selected to evaluate their cell permeability. The results show that the permeability of PAE-CD-MOF was five times higher than that of free PAE. Lung deposition tests in vivo further showed that CD-MOF loaded with PAE could be effectively transported to the deep lungs instead of being cleared directly in the airway, indicating that CD-MOF can be used as a carrier for inhaled administration [74].

### 3.5. Nanogels

Nanogels refer to three-dimensional network hydrogels with nanoscale dimensions that are formed by physically or chemically cross-linked polymers [75], with sizes ranging from 100 to 200 nm [76]. Nanogels are composed of various natural polymers, synthetic polymers, or their combinations, which facilitate the encapsulation of small molecule drugs, genes, oligonucleotides, and even proteins [39]. Nanogels, as a type of hydrogel, retain their high degree of hydration and swelling properties [39], and with their three-dimensional cross-linked network structure, they can encapsulate drugs with different chemical structures and properties. Compared to other drug delivery systems, nanogels have the advantages of easy preparation and good biocompatibility, hydrophilicity, and environmental stimulus responsiveness (such as temperature, pH, and light) [77].

Due to CD’s inherent ability to form complexes with various guest molecules and its high biocompatibility, it holds significant importance for nanogels and can be used as pharmaceutical excipients for nanogels [78]. CDs, leveraging their external hydrophilic properties, are incorporated into the polymer network of the nanogel, thereby achieving more effective drug loading and a controlled drug release [79]. For example, a novel hypoxia-sensitive supramolecular nanogel was reported in one study, which was constructed by conjugated azobenzene (Azo) and β-CD on poly (L-glutamic acid)-graft-poly (ethylene glycol) methyl ether (PLG-g-mPEG). The nanogel could efficiently load Ribonuclease A in the mild aqueous phase and achieved drug release through an azobenzene conformational transition triggered by nitroso reductase (NTR) in response to the anoxic environment of tumors [80].

Researchers have been developing innovative nanogel platforms for advanced drug delivery systems. Duan et al. developed a dual-responsive nanogel platform (HPC nanogel) for the delivery of multiple drugs. In this platform, β-CD conjugated with hyaluronic acid (HA-β-CD) and polyethyleneimine (PEI) serve as the skeleton of the nanogel, while cisplatin molecules provide internal linkage through coordination between Pt and the remaining carboxyl groups in HA-β-CD, which also possessed hyaluronidase (HAase) reactivity and glutathione (GSH) reactivity [81]. Building upon this concept, Pooresmaeil et al. designed and constructed a novel dual-responsive (pH and temperature) and photoluminescent nanogel, with carbon quantum dots (CQDs), β-CD, poly(acrylic acid) (PAA), and N-isopropylacrylamide (NIPAAm) (β-CD/NIPAM@AA) being the main components for constructing the nanogel, which was used for the delivery of methotrexate (MTX) and DOX, providing a combined drug delivery for hepatocellular carcinoma [82]. Both platforms demonstrate the versatility and adaptability of nanogels in drug delivery, highlighting progress in the field of responsive and targeted therapeutics.

### 3.6. Emulsions

Pickering emulsions are emulsions stabilized by nanosolid particles [83]. Research initiated by Ramsden and Pickering compared these with traditional emulsions and found that Pickering emulsions utilize the surface wettability of the solid particles themselves, irreversibly adsorbing the solid particles at the oil/water interface and forming a stable solid particle film to stabilize the emulsion formed by the droplets [84]. Therefore, they possess characteristics such as low toxicity [85], high security and stability [86], low cost, and environmental protection [87]. CDs can form surface-active complexes at the oil–water interface to stabilize emulsions and have potential applications due to their hydrophobic cavity and host–guest interactions [88].

Drugs encapsulated within the oil phase of oil-in-water emulsions can be protected from hydrolysis and oxidation by emulsions [89]. CDs have a unique hydrophobic cavity structure, and oil molecules and CDs can form complexes. Oil/CD complexes can prevent droplets from moving and colliding with each other (rising of oil droplets and settling of water droplets). The structural characteristics of CDs directly affect the stability of Pickering emulsions [90]. Leclercq et al. proved that natural CDs (α-, β-, or γ-CD) are highly effective in obtaining oil-in-water Pickering emulsions. Pickering emulsions obtained from the inclusion complex of oil and CD through host–guest interactions have high stability [91].

Researchers have compared the impact of HP-β-CD-based emulsions (SNEDDS) and solid dispersions on the solubility and oral bioavailability of dexibuprofen with poor water solubility. The results show that dextrobuprofen showed an amorphous form in both emulsion and solid dispersion, and the solubility of SNEDDS was significantly higher than that of solid dispersion when compared with a free drug powder. The AUC value of solid SNEDDS was significantly increased by 2.1 times and 1.6 times, respectively, leading to a marked improvement in the oral bioavailability of deibuprofen [92]. These findings suggest that CDs act as stabilizers in emulsion systems and can improve the stability and biocompatibility of emulsions.

## 4. The Application of CDs and Their Derivatives in Nano-Delivery Systems

### 4.1. Enhanced Targeting Effect

Nanoparticles, due to their specific characteristics, help to target specific areas, thereby enhancing the efficiency of delivering therapeutic drugs [93].

Colorectal cancer (CRC) is the third most common cancer worldwide, and CRC is typically associated with an inflammatory environment [94]. However, the treatment of CRC is limited. To combine therapeutic targeting with tumor microenvironment reprogramming, Bai et al. designed a biocompatible nanoparticle (CNP) that integrated regorafenib (RG) with mannitol-modified γ-CD (M-γ-CD) through host–guest complex formation to form RG@M-γ-CDCNP. The results show that the nanoparticles can reduce inflammation by targeting macrophages. It has also been shown that RG@M-γ-CDCNP exhibited targeting and biocompatibility in colitis-associated cancer and in a CT26 mouse model, which is of practical significance for adjuvant therapy in patients with CRCs [2]. In cancer, non-targeted therapies can cause toxicity and adverse side effects to normal tissues and organs. Based on this, a large amount of targeted cancer research studies are constantly developing to improve treatment efficacy and reduce side effects [95]. Baek et al. designed a kidney-removable zwitterionic CD with a customized structure for selective drug delivery in CRC. Twenty CD derivatives with different charged parts and spacers were synthesized for stability screening, and the biodistribution of five candidates was evaluated. The optimized CD exhibited higher tumor accumulation and could be used for the delivery of DOX and ulixertinib [96]. Varan et al. evaluated the in vivo efficacy and biological distribution of paclitaxel encapsulated in injectable amphiphilic CD nanoparticles with different surface charges. The results show that paclitaxel-loaded amphiphilic CD nanoparticles showed antitumor effects earlier than the drug solution. Furthermore, the trend of tumor-growth inhibition by blank nanoparticles was similar to that of the paclitaxel solution. At 24 h, the biodistribution assessed by in vivo imaging showed no accumulation of nanoparticles in the heart and lungs [97]. The above studies show that CD can be used as a carrier and can be further combined with other carrier materials (such as nanoparticles and liposomes) to develop new drug delivery systems to achieve more efficient drug targeting.

Zhang et al. successfully prepared a nano-delivery-system (Tyr/HA/CD-CS)-loaded baicalein (BA), using chitosan and β-CD (CD-CS) grafted with hyaluronic acid (HA) and D-tyrosine (D-Tyr), which were used as the raw material. The nano-delivery system could enhance the permeability of drugs to biofilms [98]. Building on this, another study developed CD-based chitosan nanospheres (CS NPs) for the nose-to-brain targeting of idebenone (IDE) to improve its low aqueous solubility and first-pass metabolism. The results show that IDE could be released slowly from CS NPs compared to free drugs. An in vitro study of bovine nasal mucosa showed that CSNPs loaded with IDE had higher permeability. The findings suggest that CD-based nanospheres have a potential applicational value in the nasal delivery of IDE for the treatment of neurological diseases [99].

Lung cancer is the tumor with the highest morbidity and mortality worldwide. Lung-targeted drug delivery technology can enrich drugs in the lungs, improve drug efficacy, and reduce side effects [100]. To address the issues of low tissue-targeting efficiency and severe side effects in pulmonary drug delivery, based on the lung-targeting characteristics of cubic cross-linked CD metal–organic framework (CDF) nanoparticles, He et al. utilized RGD-functionalized CDF to deliver low-molecular-weight heparin (LMWH) and DOX to treat lung cancer. The results show that the nanoparticles could effectively target lung tumors after intravenous administration, and accumulation in the lung was 5.8 times higher than that in the liver [101].

### 4.2. Regulation of Drug Release

Compared with traditional drug delivery systems, CD-based nano-delivery systems can achieve a sustained release of drugs. Matshetshe et al. developed β-CD-modified chitosan nanoparticles (β-CD/CS-NPs) as carriers for cinnamon essential oil (CEO), which is known for its volatility and relatively low stability. The research findings indicate that these nanoparticles were spherical, and they achieved an encapsulation efficiency of 58% for CEO at 55 °C, which was significantly higher than that of CEO-loaded CS nanoparticles (approximately three times as much). Additionally, in vitro release studies demonstrated that the release of CEO from β-CD/CS-NPs was sustained and controllable for over 120 h [102].

CD has the characteristics of low toxicity, good biocompatibility, and ease of modification, so it is widely used in stimulus-responsive systems [103]. For example, Li et al. based the oral delivery of doxorubicin hydrochloride and celastrol (CSL) on mono-(6-pentethylenehexamine)-β-CD (PEHA-β-CD) and sodium dodecylbenzene sulfonate (SDBS) to pH-responsive supramolecular nanoparticles (PEHA-β-CD/SDBS) through electrostatic interactions. These nanoparticles minimally released the drugs in an acidic pH environment (such as the stomach at pH = 1.2) and effectively released them in an alkaline pH environment (such as the intestine at pH = 8.5), making them suitable as carriers for oral drug administration [104]. Unlike normal cells, cancer cells have a higher metabolic rate and a faster proliferation speed, which makes the tumor tissue form a unique microenvironment. The tumor microenvironment has several significant pathological characteristics, such as a lower pH value, EPR effect (enhanced permeability and retention effect), and hypoxia [105]. Mrówczyński et al. functionalized polydopamine-coated DOX magnetic nanoparticles with mono-6-thio-β-CD (SH-β-CD). The encapsulation efficiency of this nano-system could reach up to 90%, and the cumulative release of DOX within 10 h was approximately 9% at a pH of 5.5 and 11% at a pH of 4.5. After nearly 50 h, the release of DOX reached its maximum value. This pH-sensitive release property matches the acidic characteristics of the tumor microenvironment, indicating that this nano-system could serve as an ideal carrier material for cancer treatment [106]. Ramasamy et al. used a CD–dextran complex to coat nickel ferrite nanoparticles and improved their antitumor effect by loading camptothecin, with a loading rate of the nanocarrier reaching 88%. The in vitro release curve showed that the drug was stable and sustained for more than 500 h, and a decrease in pH from 7.4 to 6.0 would cause the drug to be released faster. It was further confirmed that the nanocarrier was suitable for transporting anti-cancer drugs and could respond to pH changes in the tumor microenvironment [107]. These studies indicate that CD-based nanocarriers can achieve a controlled and sustained drug release in the tumor microenvironment through their pH sensitivity, providing effective strategies and materials for cancer treatment.

### 4.3. Improving Drug Properties

Improving the properties of drugs includes increasing drug stability, solubility, and bioavailability. As pharmaceutical excipients, CDs are often used to enhance the water solubility of poorly soluble drugs, increase the permeability of drugs through biological membranes, and thus improve the bioavailability of drugs [108].

Due to the susceptibility of intracellular ROS and immunotherapeutic drugs to degradation in vivo, effectively delivering genes or small-molecule drugs to macrophages remains a challenge. Cheng et al. employed star amphiphilic biocompatibility β-CD-graft-(poly(ε-caprolactone)-block-poly-(2-(dimethylamino)ethyl methacrylate)_x_ (β-CD-g-(PCL-b-PDMAEMA))_x_) copolymers. Through the interaction between genes and cationic PDMAEMA blocks, genes were delivered to macrophages, in which the participation of poly(ε-caprolactone) fragments (PCLs) could enhance the stability of the copolymer by micelle formation, thus significantly improving gene transfection efficiency by up to 10.8% [109].

The water solubility and oral bioavailability of BCS II and IV drugs can be enhanced by complexation with CDs, where Gidwani et al. studied the formation of inclusion complexes, and the results show that the solubility of the inclusion complex was significantly higher than that of the free drug, with the dissolution volume reaching nearly 50% within 45 min. This rapid dissolution was due to the improved wettability of CD and its rapid formation in the solution and its soluble complex ability [110]. To improve the water solubility of resveratrol (RES), Wang et al. prepared RES and a sulfobutyl ether–β-CD complex (CD-RES) and loaded it into polymer nanoparticles. The results show that the nano-carrier increased the water solubility of RES by 66 times. In addition, it also showed a better anticancer effect [111].

Rilpivirine belongs to the BCS II class of drugs and is used to treat HIV infections. Rao et al. studied the use of β-CD-based nano-sponges to improve the solubility of rilpivirine [112]. It has been found that the microwave-assisted synthesis of β-CD nano-sponges can improve the solubility and drug delivery potential of domperidone, thereby improving its oral bioavailability [113].

Nano-sponges have a long stability period when in powder form, which can prevent the degradation of the drug molecules loaded inside [114]. Sharma et al. encapsulated ellagic acid (EA) into CD nano-sponges (CDNSs), and compared to distilled water, the solubilization effect of CDNSs increased by 10 times. In addition, the photostability of EA significantly improved [115].

CD-MOFs can load different drug molecules in their extended framework through co-crystallization [116], and the drug loading of dense metal–organic frameworks (MOFs) can be increased by converting it into a porous form. For example, potassium acetate γ-CD metal–organic frameworks (γ-CD-MOFs) were transformed into a porous form by ethanol to improve their drug-loading capacity [117]. CD-based nanoliposomes have been proven to enhance the bioavailability and stability of drugs such as butylphthalide [118], anethole [119], and linalool [66]. Through these studies, we find that CD and its derivatives have wide applications in improving the water solubility, stability, and bioavailability of drugs, providing a variety of effective strategies in the field of nano-delivery systems.

### 4.4. Improved Drug-Loading Efficiency

Most nanomedicines have a relatively low drug-loading capacity, which can lead to insufficient drug release and can hinder the clinical translation of nanomedicines. Therefore, it is crucial to improve the drug-loading and encapsulation efficiency of nanomedicines. Not only can this reduce the potential adverse effects of excessive nanomaterials, but it can also lower the manufacturing costs of nanomedicines [120].

As drug carriers, CDs have attracted extensive attention in nano-delivery systems because of their ability to improve the drug encapsulation efficiency and drug load. Gaetano et al. developed sulfobutylether-β-CD-based chitosan nanoparticles (CH/SBE-β-CDNPs) for the ocular delivery of levofloxacin (LVF). Due to the complexation of LVF with SBE-β-CD, both the encapsulation efficiency and drug load were significantly improved compared to free LVF, from 21.53% ± 1.47 and 25.33% ± 1.24 to 41.50% ± 1.19 and 47.83% ± 2.20, respectively, and the bactericidal activity increased by approximately 2 times. The results suggest that CHNPs based on SBE-β-CD may be a potential delivery system for LVF in the treatment of eye infections [121]. In addition, a study by Varan et al. further confirmed the effect of CD type on the drug-loading efficiency of nanosphere. They found that the drug-loading efficiency of nanospheres had a wide range (between 49% and 87%), and the encapsulation efficiency increases in the order of 6OCaproβCD < 6OCaproαCD < PC βCDC6 [122].

The entrapment efficiency of hydrophobic drugs based on liposomes usually depends on the mass ratio of the drug to the liposome. A large number of hydrophobic drug molecules will destroy the integrity and stability of the bilayer structure of liposomes [123]. The water solubility of the drug increases after forming an inclusion complex with the CD, and the complex can be loaded into the liposomes, thus increasing the drug loading of the insoluble drug. Wang et al. encapsulated brinzolamine and hydroxypropyl-β-CD into nanoliposomes (BCLs), achieving an encapsulation efficiency of 92.50 ± 2.1% and showing continuous release [124].

CD-based nano-sponges have been demonstrated to be promising drug carriers, as CD can enhance the drug encapsulation efficiency, thereby improving drug stability and solubility [49]. Dhakar et al. achieved a high drug-loading (19.06%) and -entrapment (95.31%) efficiency using CD nano-sponge-loaded kurenic acid [125]. In addition, Mendes et al. developed a norfloxacin nano-sponge with a high encapsulation efficiency (80%) based on CD to improve its physical and chemical properties and to promote oral absorption, mainly by changing β-CD and the cross-linking agent diphenyl carbonate to improve the encapsulation efficiency [126].

The applications and functions of CDs and their derivatives in nano-delivery systems such as nanospheres, nano-sponges, and CD metal–organic frames are summarized in Table 2.

## 5. Conclusions and Prospects

CDs are a kind of cyclic oligomer of glucose, which have excellent biocompatibility and low immunogenicity. In host–guest chemistry, CDs can wrap suitable hydrophobic guest molecules or ions in their cavity. Compared with covalent bonds, this non-covalent interaction is cost-effective and environmentally friendly [134], so CD-based polymers have a high specific surface area and good biocompatibility and biodegradability. For example, as a new green material, CD-based eutectic supramolecular polymer plays an important role in the encapsulation and solubilization of compounds [135].

This review summarized the main research methods involved in novel drug delivery systems based on CDs and their derivatives as nanocarriers, including nanospheres, nano-sponges, CD metal–organic frameworks, nanogels, liposomes, and emulsions. CDs can form host–guest complexes with a variety of hydrophobic guest molecules. This unique structural property gives CDs and their derivatives a broad applicational prospect in drug delivery systems, playing an important role in targeted drug delivery, regulating drug release, and improving drug delivery characteristics and bioavailability. They are expected to play an even more significant role in future drug delivery applications. Although CDs have great potential in nano-delivery systems, there are also some challenges, such as the solubility, stability, and biodegradability of CDs, as well as their interactions with drug molecules, and the long-term stability, safety, and efficacy of these formulations still require further clinical trials for validation. Therefore, future research needs to further optimize the chemical structure and functionality of CDs to achieve a more efficient and safer drug delivery. At the same time, the combination of two or more nano-delivery systems may fully exert the advantages of each drug delivery system, forming an excellent composite drug delivery system, further improving the physicochemical properties of drugs, and thereby better exerting the clinical therapeutic effect.

## Figures and Tables

**Table 1:** Characteristics of α-CD, β-CD, and γ-CDs [22,23,24].

Property	α-CD	β-CD	γ-CD
Molecular formula	C_36_H_60_O_30_	C_42_H_70_O_35_	C_48_H_80_O_40_
Number of glucose units	6	7	8
Molar mass (g/mol)	972.85	1134.99	1297.13
Solubility in water at room temperature (mg/mL)	129.5 ± 0.7	18.4 ± 0.2	249.2 ± 0.2
Moisture content (%*w*/*w*)	10.2	13.0–15.0	8–18

**Table 2:** The role of different CDs in nano-delivery systems.

Nano-Delivery System	CD Type	Size/nm	Active Ingredients	Effect	References
Nanoparticles	HPCD-HMD	120–200	Meropenem	Improved the solubility of drugs in aqueous solutions.	[127]
	Mannose-modified γ-CD	100–300	Regorafenib (RG)	Improved the biodistribution and pharmacokinetic and pharmaceutical properties of RG.	[2]
	6OcaproβCD and PC βCDC6	113 ± 4 and 82 ± 2	Paclitaxel	Improved the antitumor effect.	[97]
Nanogels	HP-β-CD	310.65 ± 18.75	Dexibuprofen	This nanogel, which has porous and amorphous shapes, can significantly enhance drug release, and the formulation demonstrated good biocompatibility.	[128]
	β-CD-conjugated hyaluronic acid (HA-βCD)	36.0 ± 4.5	Small molecules and proteins	The HPC nanogels were a robust and universal drug delivery nanoplatform.	[81]
	β-CD	657	Methotrexate (MTX) and doxorubicin (DOX)	These nanogels were double-responsive (pH and temperature) and photoluminescent.	[82]
Nanospheres	Am-CD/RNA	390	SiRNA/sorafenib	The nanogel achieved synergistic therapy for hepatocellular carcinoma.	[44]
	HP-β-CD	140	Idebenone (IDE)	There was a higher permeation/interaction of IDE-loaded CS NPs with respect to free IDE.	[99]
	α- and β-CD	88–270	Erlotinib (ERL)	The nanospheres could increase ERL’s anticancer efficacy with conventional and 3D tumor models made in lung and hepatocellular carcinoma cells.	[122]
Nano-sponge	β-CD-CMC-g-poly	195–250	Docetaxel	The water solubility of docetaxel significantly improved (by up to 14 times).	[129]
	β-CD	51.38–154.56	Tapentadol	The drug release rate in 6 h was 51.62–82.34%, which significantly improved the controlled-release ability.	[130]
MOFs	γ-CD	The mean pore size of CD-MOFs is 1.4 nm.	Paeonol (PAE)	The permeability of PAE-CD-MOF was 5 times higher than that of free PAE.	[74]
	γ-CD	200–500	Honokiol (HNK)	The MOF improved the solubility and dissolution rate of HNK.	[131]
Liposome	E-βCD/D-βCD/βCD	146–163	Curcumin	The encapsulation efficiency of liposome was more than 5 times higher than that of normal liposome.	[132]
	HP-β-CD	82.29 ± 6.20	Brinzolamide (BRZ)	The liposome had an entrapment efficiency (EE) of 92.50 ± 2.10%.	[124]
Emulsion	γ-CD/sodium caseinate/alginate (Alg)	138 ± 6 and 206 ± 12	Curcumin	The liposomes were stable under the conditions of high acidity (pH 3.0), high alkalinity (pH 11.0), and high temperature (90 °C).	[133]
	α-CDs were modified with octenylsuccinic anhydride (OSA)	10–100 μm	Curcumin	It possessed good storage stability after 30 days of storage. In addition, emulsion with a smaller particle size had a higher free fatty acid release and increased bioavailability by 10.3%.	[122]
